# Assessment of level of knowledge, attitude, and associated factors toward delirium among health professionals working in intensive care unit multicenter, cross-sectional study, Amhara region comprehensive specialized hospitals, Northwest Ethiopia, 2023

**DOI:** 10.3389/fpubh.2024.1338760

**Published:** 2024-03-06

**Authors:** Ruth Ayanaw Eyayu, Tadael Gudayu Zeleke, Wubie Birlie Chekol, Debas Yaregal Melesse, Henos Enyew Ashagrie

**Affiliations:** Department of Anaesthesia, College of Medicine and Health Sciences, University of Gondar, Gondar, Ethiopia

**Keywords:** attitude, delirium, intensive care, knowledge, professionals

## Abstract

**Background:**

Patients in Intensive Care Unit (ICU) are at high risk of developing delirium. Lack of early detection and the inability to provide prompt management of delirium remain challenges of ICU patient care. This study aimed to assess the level of knowledge, attitude, and associated factors toward delirium among healthcare providers working in ICU.

**Methods:**

A multicenter, cross-sectional survey was conducted in comprehensive specialized hospitals from 15 April to 5 June 2023. Data were collected using a pretested, self-administered questionnaire. Ordinal logistic regression analysis was performed at *p* < 0.05 with a 95% confidence interval (CI). The odds ratio with 95% CI was calculated to determine the strength of the association between independent and outcome variables.

**Results:**

A total of 202 health professionals were included in this study, with a response rate of 87%. The proportions of good, moderate, and poor knowledge about delirium in ICU were 29.21 (95% CI: 23–36), 52.48 (95% CI: 45.3–59.5) and 18.32 (95% CI:13.2–24.4), respectively. The overall proportion of negative, neutral and positive attitude were 13.9 (95% CI: 9.4–19.4), 65.8 (95% CI: 58.9–72.4) and 20.3 (95% CI: 15–26.5) respectively. Being an anesthetist and exposure to training were positively associated with a good knowledge while belief in screening tool to change care and reading, and using guidelines were positively associated with a positive attitude. However, believing the impossibility of changing the practice of delirium care, and negative attitudes were delaying factors for a good knowledge. Also, workload and poor knowledge were hindering factors for a positive attitude.

**Conclusion:**

More than half of health professionals had moderate knowledge and neutral attitude toward delirium. However, some of them had poor knowledge and a negative attitude. We recommend stakeholders prepare regular training for delirium care. Also, we urge health professionals to update themselves by reading guidelines and to use screening protocols for delirium.

## 1 Introduction

Delirium is defined as an acute confounding state with a complex clinical syndrome characterized by disturbance of consciousness, attention, cognitive function, or perception that develops within a short period of time and tends to fluctuate during the course of the day (1). It is a common incidental problem in intensive care unit (ICU), which accounted for 68% in India (2); 51% in Uganda (3); 80% in USA (4) and 22.9% in Mexico, of which 12% of the victims ended up with death (5). Delirium would be high in older patients, immobilized patients, patients on multiple medications, patients with sepsis, medical illnesses, and those on mechanical ventilation (6–8). Even though delirium is usually transient and a reversible syndrome, when left untreated, it will expose patients to prolonged hospital stays, prolonged mechanical ventilation, long-term cognitive dysfunction, and increased mortality (6, 9). The incidence and duration of delirium in ICU patients could be effectively reduced by using multicomponent interventions such as physical activity, family participation, cognitive stimulation, reorientation, sensory stimulation, environmental control, clinical adjustment, and reorientation (10). Assessment of the patient’s conscious level and use of validated detection tools are paramount for the diagnosis and treatment of delirium (11).

However, this condition is often underrecognized and not addressed effectively (12).

As knowledge of delirium and its management approach has changed significantly, professionals those are unaware of it might have a poor level of knowledge, and bad attitude, and poor practice on their patient care (13). Studies also reported that health care providers frustrated to provide care for delirium patients, with arguable reasons of lack of confidence on assessment and dislike of delirium management (14). Reports showed patients with delirium being viewed as underestimated, ignored, and considered a “burden.” This can lead to negative outcomes for patients, hospital staff, and the system of health care because of increased hospitalization and the need for expensive interventions in the event of complications (15). Problems in early detection and management of delirium by health professionals working in the ICU has been reported (2). Lack of knowledge on diagnosis and screening tools, poor attitudes toward delirium care, communication challenges, time restraints, and workload were among the barriers to poor detection and management of delirium (9). The use of validated assessment tools can ensure early detection and the provision of appropriate care to achieve good outcomes (16). To the best of our knowledge, there is no previous study that determines health professionals’ knowledge, attitude, and barriers toward the diagnosis and monitoring of delirium in the study region. It is well understood that the use of updated assessment tools for the measurement of delirium relies on health care providers’ beliefs, perceptions, and attitudes toward delirium. In the present study, we carried out a multi-center survey involving critical care physicians, non-physician anesthetists and ICU nurses regarding their current knowledge and attitude of delirium as well as perceived barriers to the screening and monitoring of delirium. The results of our study may be used us a source for future researchers, and it can also help policymakers to restructure the working environment and improve the management of delirious patients.

## 2 Methods

A hospital-based, multicenter cross-sectional study was followed. The study was conducted on surgical and medical ICUs at four comprehensive specialized hospitals located in Amhara National Regional State, north-west Ethiopia. The duration of the research was from 15 April to 5 June 2023. Before data collection, we communicate with the ICU leaders of the four hospitals to ensure the homogeneity of the study participants. We found 232 eligible study participants working in almost similar surgical and medical ICU setups.

Inclusion criteria:

- Physicians (Internists, surgeons, surgical and medical residents involved in ICU patient care).
- Anesthetists/Anesthesiologist (Anesthesia and critical care providers working in the ICUs).
- ICU nurses (Intensive care specialist nurses working in the ICUs).

Part-time workers, volunteers, and carers on leave during the data collection period were excluded from the study. Sociodemographic variables, cultural variables, and work-related variables were included in the self-administered questionnaire. Levels of knowledge and attitude were the main outcome variables. Knowledge was defined as the familiarity or awareness of someone or something, such as facts, skills, or objects contributing to understanding. Overall knowledge score was categorized using Bloom’s cut-off points for KAP study 80%–100% for good knowledge 60%–79% for moderate knowledge, and ≤59% for poor knowledge (17). Attitude was defined as critical care providers’ thinking and feeling about the diagnosis and the clinical significance of delirium. A total of 22 items were included in the questionnaire, which was subdivided into three domains (emotion, behavior, and belief). It was measured using five point-Likert scale (from completely disagree to completely agree). The overall attitude score was categorized using Bloom’s cut-off points for KAP study 80%–100% for positive attitude, 60%–79% for neutral attitude, and ≤59% for negative attitude (17). Many studies adapted this cutoff point to determine the level of knowledge, attitude, and practice for their respective studies (18–22).

In this survey, the total study population in the study areas was 232 and easily accessible, so we took the whole population in the four aforesaid hospitals.

After completion of data collection, the variables were entered, coded, and cleaned for errors using Epi-data software. Then the data were transformed into SPSS version 25 software and analyzed. Descriptive data were expressed with frequency and percentage. The Shapiro–Wilk test was checked for the distribution of data. Non-normally distributed data were presented with a median and interquartile range. The model goodness of fit test, multi-collinearity test and parallel line test were checked.

Ordinal logistic regression analysis was conducted to assess the association between dependent and independent variables. The odds ratio with the corresponding 95% CI was calculated to determine the strength of the association of the independent factors with outcome variables. A *p*-value < 0.05 was considered as statistically significant predictive factors. Finally, the results were presented with texts, tables, and graphs.

Ethical clearance was obtained from Ethical Review Committee of the respective hospitals. Informed consent was obtained from each study participant after a clear explanation of the merits of the study, and confidentiality and anonymity were ensured.

## 3 Results

### 3.1 Socio-demographic characteristics

Among 232 eligible study participants, 202 of them were included in the final analysis with a response rate of 87%. Fifty (24.1%) physicians, 77 (38.1%) anesthetists and 75 (37.1%) nurses participated in the study. Thirty study subjects were excluded from the analysis: nine due to incomplete data and 21 due to refusal to fill out the data. Among participants, 147 (72.8%) were males, while 55 (27.2%) were females. The median age of the study participants was 31 (28.75–35). The majority of the health professionals had work experience of 5 to 9 years in their profession (Table 1).

**Table 1:** Socio-demographic characteristics of health professionals working in ICU, Amhara Region Comprehensive Specialized Hospitals, Northwest Ethiopia, 2023, (*N* = 202).

Variables	Frequency	Percentage
**Age category**		
25–34 years	145	71.8%
35–44 years	57	28.2%
**Gender**		
Male	147	72.8%
Female	55	27.2%
**Profession**		
Nurse	75	37.1%
Anesthetist	77	38.1%
Physician	50	24.7%
**Educational level**		
Bachelor	66	32.7%
Masters and above	86	42.6%
Resident	32	15.8%
General practitioner	11	5.4%
Specialist	7	3.5%
**Work experience**		
1–4 years	79	39.1%
5–9 years	85	42.1%
≥10 years	38	18.8%
**Experiences in ICU**		
<1 years	66	32.7%
1–4 years	108	53.5%
5–9 years	22	10.9
≥10 years	6	3.0%

### 3.2 Work-related characteristics and beliefs of health professionals

Most, 172 (85.1%) of the study subjects acknowledged the usefulness of a screening tool for delirium while 165 (81.7%) of them had no prior experiences at institutions where protocols or guidelines were implemented.

The overall median responses to knowledge and attitude questions were 73 (63–80) and 71 (64–78), respectively.

### 3.3 Level of attitude and knowledge across hospitals

**Figure 1:**
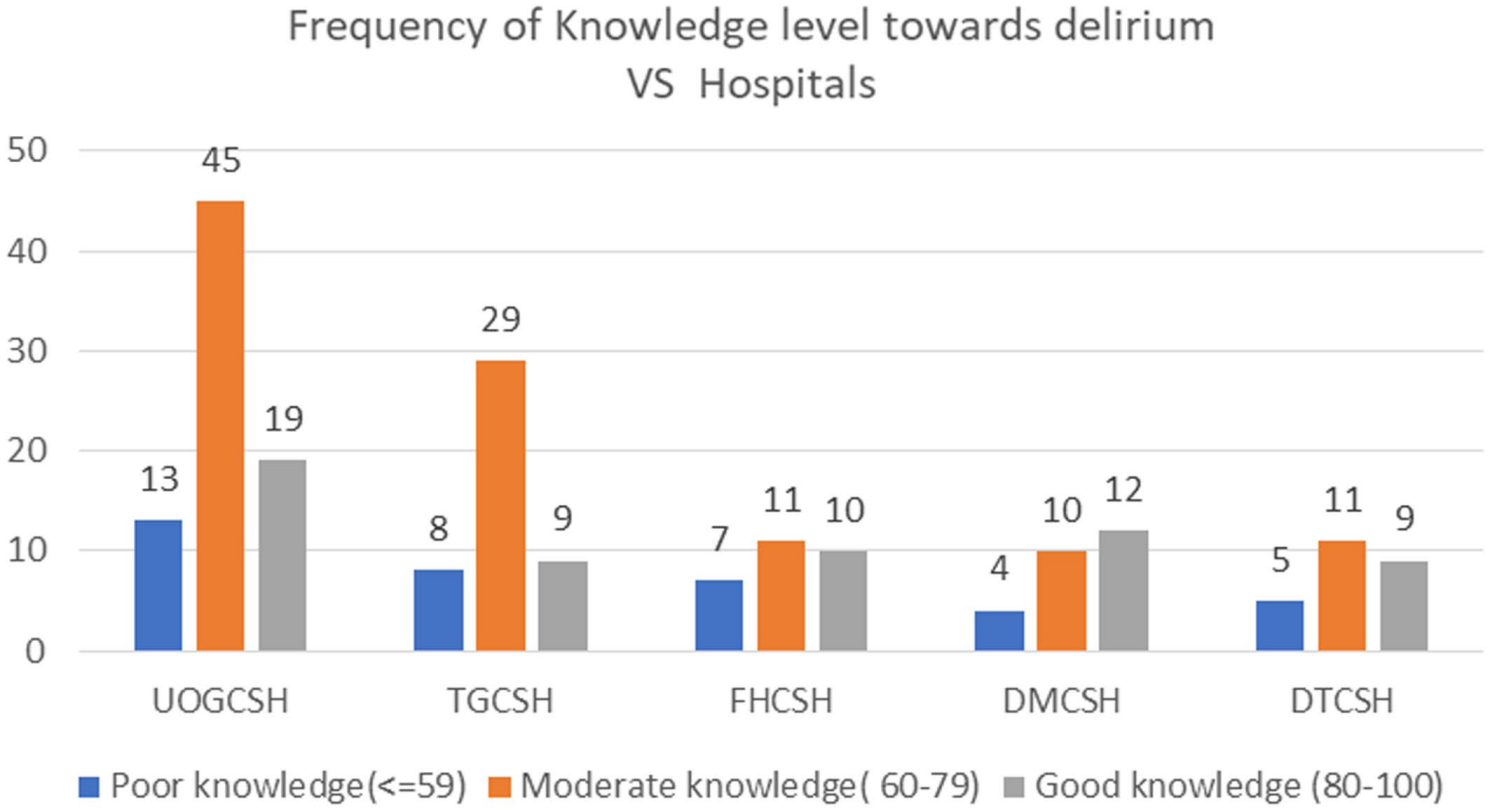
Frequency of Knowledge level toward delirium vs. Amhara Region Comprehensive Specialized Hospitals, Northwest Ethiopia, 2023, (*N* = 202).

**Figure 2:**
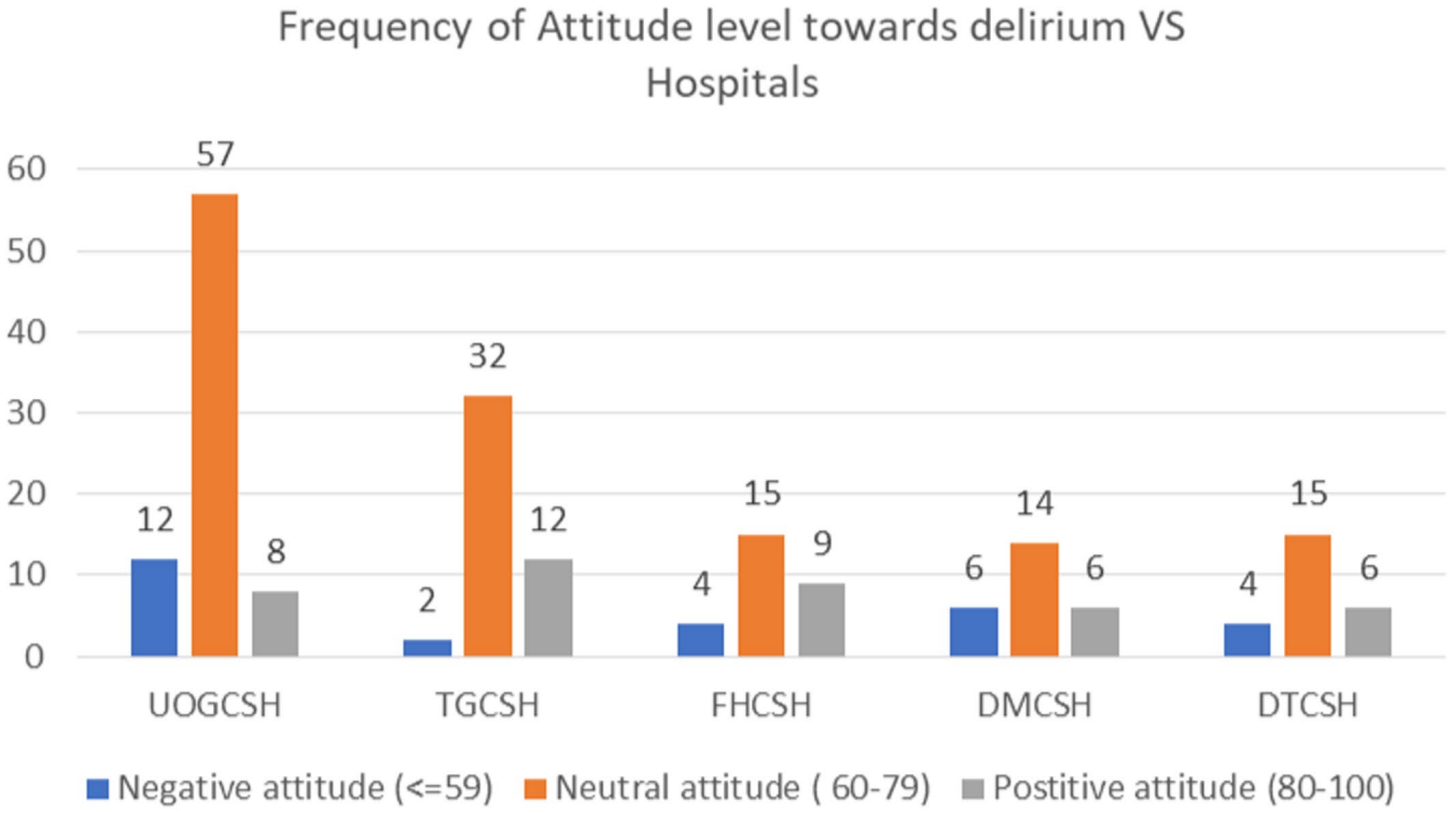
Frequency of Attitude level toward delirium vs. Amhara Region Comprehensive Specialized Hospitals, Northwest Ethiopia, 2023, (*N* = 202).

In the University of Gondar comprehensive specialized hospital (UOGCSH), 45 (22.2%) study subjects had moderate knowledge, while 19 (9.4%) of them had good knowledge (Figure 1). In Tibebe Ghion Comprehensive Specialised Hospital (TGCSH), 32 (15.8%) study subjects had neutral attitude, whereas 12 (5.9%) had a positive attitude (Figure 2).

### 3.4 Overall proportions of knowledge and attitude toward delirium

**Figure 3:**
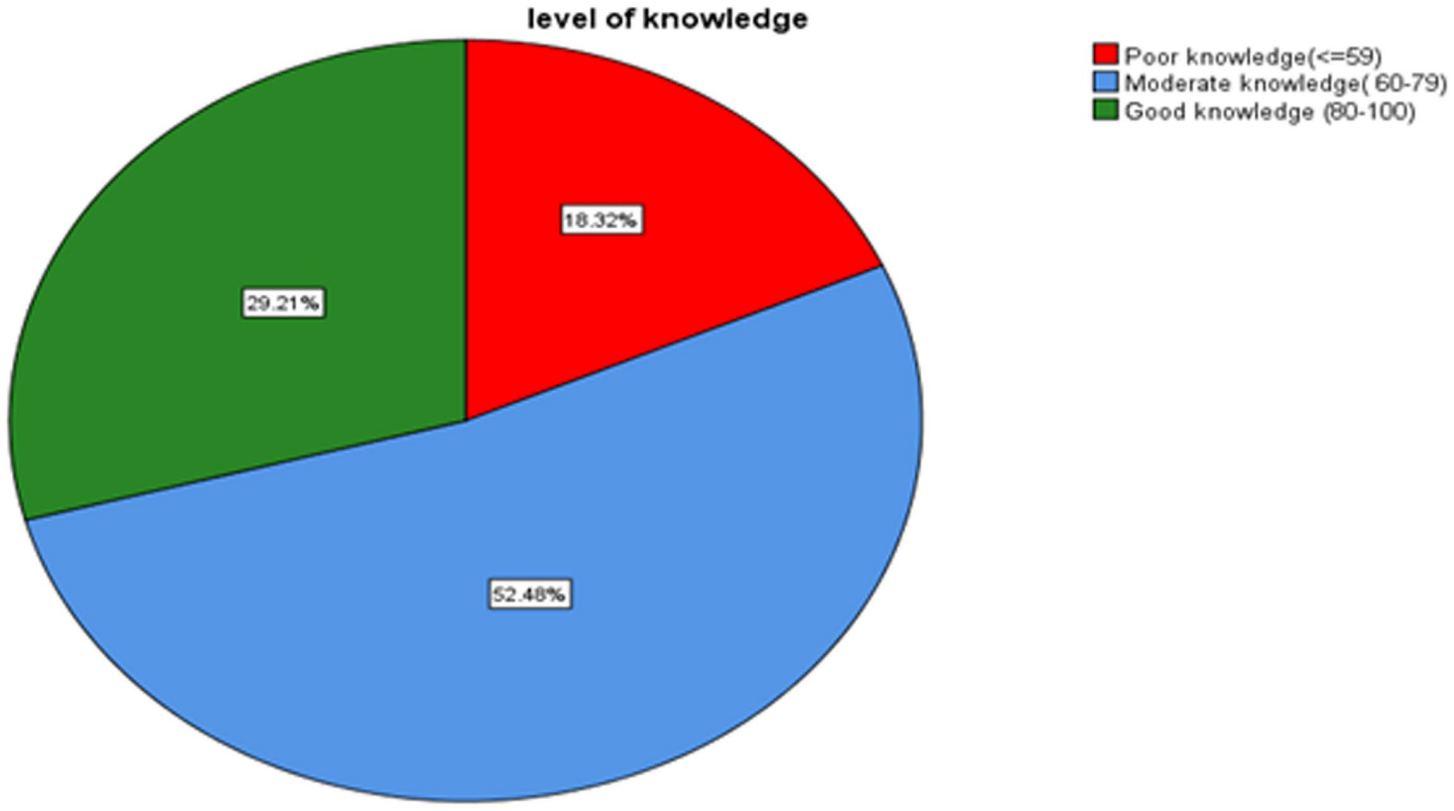
Proportions of knowledge level toward delirium among health professionals working in ICU, Amhara Region Comprehensive Specialized Hospitals, Northwest Ethiopia, 2023, (*N* = 202).

**Figure 4:**
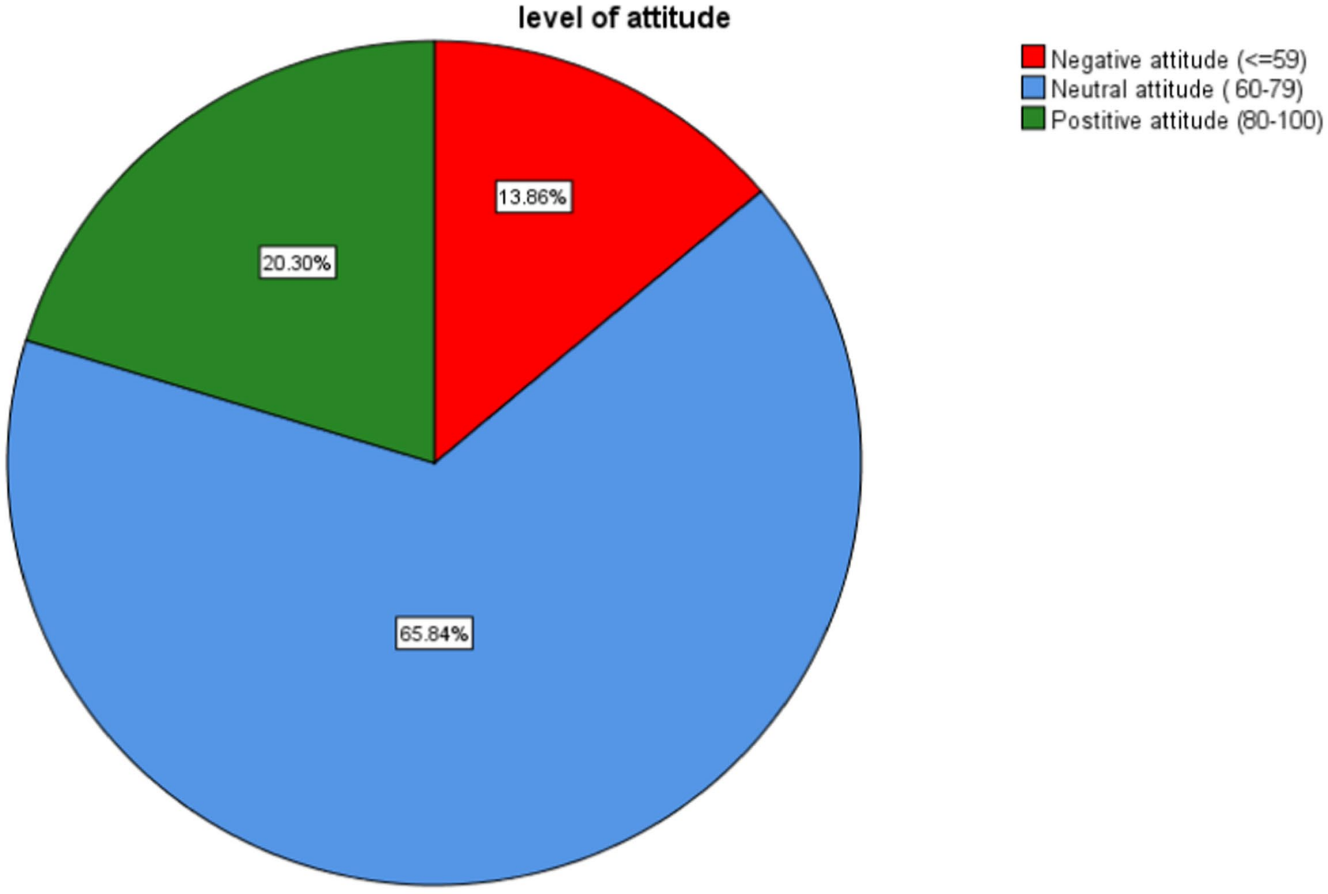
Proportions of attitude level toward delirium among health professionals working in ICU, Amhara Region Comprehensive Specialized Hospitals, Northwest Ethiopia, 2023, (*N* = 202).

The overall proportion of good, moderate, and poor knowledge was 29.21 (95% CI: 23–36), 52.48 (95% CI: 45.3–59.5), and 18.32 (95% CI: 13.2–24.4), respectively (Figure 3). The overall proportion of negative, neutral, and positive attitudes was 13.9 (95% CI: 9.4–19.4), 65.8 (95% CI: 58.9–72.4) and 20.3 (95% CI:15–26.5) respectively (Figure 4).

### 3.5 Factors associated with knowledge level toward delirium

Factors showing an association with knowledge in bivariable ordinal logistic regression analysis were entered into multivariable ordinal logistic regression. Anesthetists were more likely to have good knowledge as compared to physicians holding all other variables constant (AOR = 2.8, 95% CI =1.25–6.26, *p* = 0.012). Believing the possibility of changing the practice of delirium care was less likely to have good knowledge than believing possibility of change (AOR = 0.48, 95% CI =0.24–0.97, *p* = 0.041).

Health professionals engaged in training about delirium were more likely to have good knowledge as compared to those lacking the training (AOR = 2.16, 95% CI =1.14–4.1, *p* = 0.018). The odds of having a negative attitude were less likely to have good knowledge than having a positive attitude (AOR = 0.06, 95% CI = 0.02–0.19, *P* ≤ 0.001).

Professionals with neutral attitude were less likely to have good knowledge than having positive attitude (AOR = 0.13, 95% CI = 0.05–0.29, *P* ≤ 0.001; Table 2).

**Table 2:** Ordinal logistic regression for knowledge level toward delirium among health professionals working in ICU, Amhara Region Comprehensive Specialized Hospitals, Northwest Ethiopia, 2023, (*N* = 202).

Variables	Knowledge level			Odds ratio with 95%CI		*p*-value
				COR	AOR	
	Poor *N* (%)	Moderate *N* (%)	Good *N* (%)			
**Gender**						
Male	19(9.4)	81(40.1)	47(23.3)	2.37(1.28–4.4)	1.81(0.87–3.76)	0.111
Female	18(8.9)	25(12.4)	12(5.9)	1	1	1
**Profession**						
Nurses	21(10.4)	40 (19.8)	14(6.9)	0.52(0.13–1.03)	1.73(0.74–4.02)	0.204
Anesthetists	10(5)	35(17.3)	32(15.8)	1.6(0.81–3.14)	**2.8(1.25–6.26)**	**0.012**
Physicians	6(3)	31(15.3)	13(6.4)	1	1	1
**Believing that delirium screening tool will change delirium care**						
Yes	27(13.4)	88(43.6)	57(28.2)	5.44(1.63–7.26)	1.01(0.42–2.45)	0.975
No	10(5)	18(8.9)	2(1)	1	1	1
**Believing that delirium is not preventable**						
Yes	14(6.9)	19(9.4)	7(3.5)	0.37(0.18–0.71)	0.63(0.28–1.4)	0.254
No	23(11.4)	87(43.1)	52(25.7)	1	1	1
**Believing that it is impossible to change the practice of delirium care**						
Yes	13(6.4)	32(15.8)	4(2)	0.33(0.18–0.62)	**0.48(0.24–0.97)**	**0.041**
No	24(11.9)	74(36.6)	55(27.2)	1	1	1
**Prior experience in an institution where protocols/guidelines used to screen delirium**						
Yes	3(1.5)	17(8.4)	17(8.4)	2.57(1.29–5.14)	2.05(0.9–4.69)	0.087
No	34(16.8)	89(44.1)	42(20.8)	1	1	1
**Received delirium related courses educations/trainings**						
Yes	6(3)	47(23.3)	35(17.3)	3.07(1.76–5.37)	**2.16(1.14–4.10)**	**0.018**
No	31(15.3)	59(29.2)	24(11.9)	1	1	1
**Read guidelines/protocols about delirium**						
Yes	8(4.0)	52(25.7)	35(17.3)	2.55(1.48–4.41)	1.19(0.62–2.28)	0.597
No	29(14.4)	54(26.7)	24(11.9)	1	1	1
**Level of attitude**						
Negative attitude	12(5.9)	15(7.5)	1(0.5)	0.03(0.01–0.09)	**0.06(0.02–0.19)**	**<0.001**
Neutral attitude	25(12.4)	78(38.6)	30(14.9)	0.12(0.06–0.26)	**0.13(0.05–0.29)**	**<0.001**
Positive attitude	0(0.0)	13(6.4)	28(13.9)	1	1	1
**Poor working collaboration between health professionals**						
Yes	20(9.9)	51(25.2)	45(22.3)	2.1(1.23–3.60)	1.33(0.71–2.48)	0.379
No	17(8.4)	55(27.2)	14(6.9)	1	1	1

The bold values indicates the *p*-value less than 0.05.

### 3.6 Factors associated with attitude level toward delirium

Factors showing an association with attitude in bivariable ordinal logistic regression analysis were entered into multivariable ordinal logistic regression. Believing that the screening tool will change delirium care was more likely to have a positive attitude as compared to those who did not believe, holding all other variables constant (AOR = 3.71, 95% CI =1.35–10.15, *p* = 0.011). The estimated odds ratio (AOR = 2.7, 95% CI =1.23–5.92, *p* = 0.013) indicated that health professionals who use protocols or guidelines to screen delirium were more likely to have a positive attitude than those who do not use them.

Reading updated guidelines and protocols was more likely to have a positive attitude as compared to not reading (AOR = 2.55, 95% CI =1.17–5.59, *p* = 0.019). Professionals with overloaded work were less likely to have a positive attitude when compared to those with a lower workload (AOR = 0.41, 95% CI = 0.17–0.96, *p* = 0.04). Working in the surgical ICU was less likely to have a positive attitude than working in the mixed ICU (AOR = 0.41, 95% CI = 0.18–0.96, *p* = 0.04). Having poor knowledge was less likely to a have a positive attitude than having good knowledge (AOR = 0.04, 95% CI = 0.01–0.13, *P* ≤ 0.001). Having a moderate knowledge was times less likely to have positive attitude than having a good knowledge (AOR = 0.15, 95% CI = 0.06–0.36, *P* ≤ 0.001; Table 3).

**Table 3:** Ordinal logistic regression for attitude level toward delirium among health professionals working in ICU, Amhara Region Comprehensive Specialized Hospitals, Northwest Ethiopia, 2023, (*N* = 202).

Variables	Attitude level			Odds ratio with 95% CI		*p-*value
				COR	AOR	
	Negative *N* (%)	Neutral *N* (%)	Positive *N* (%)			
**Gender**						
Male	17(8.4)	98(48.5)	32(15.8)	1.65(0.86–3.16)	0.57(024–1.32)	0.189
Female	11(5.4)	35(17.3)	9(4.5)	1	1	1
**Profession**						
Nurses	15(7.4)	51(25.2)	9(4.5)	0.3(0.14–0.65)	0.48(0.17–1.32)	0.155
Anesthetists	8(4.0)	54(26.7)	15(7.4)	0.56(0.27–1.18)	0.54(0.21–1.4)	0.203
Physicians	5(2.5)	28(13.9)	17(8.4)	1	1	1
**Believing that delirium screening tool will change delirium care**						
Yes	16(7.9)	115(56.9)	41(20.3)	7.71(3.31–17.94)	**3.71(1.35–10.15)**	**0.011**
No	12(5.9)	18(8.9)	0(0.0)	1	1	1
**Believing that delirium is not preventable**						
Yes	13(6.4)	21(10.4)	6(3.0)	0.32(0.15–0.68)	0.61(0.24–1.55)	0.300
No	15(7.4)	112(55.4)	35(17.3)	1	1	1
**Believing that it is impossible to change the practice of delirium care**						
Yes	15(7.4)	27(13.4)	7(3.5)	0.32(0.16–0.65)	0.74(0.31–1.73)	0.481
No	13(6.4)	106(52.5)	34(16.8)	1	1	1
**Types of ICU**						
Medical ICU	3(1.5)	20(9.9)	1(0.5)	0.44(0.18–1.08)	1.06(0.33–3.36)	0.924
Surgical ICU	10(5.0)	36(17.8)	7(3.5)	0.48(0.24–0.94)	**0.41(0.18–0.96)**	**0.04**
Mixed (Medical-Surgical)	15(7.4)	77(38.1)	33(16.3)	1	1	1
**Presence of protocols/guidelines to screen delirium**						
Yes	3(1.5)	43(21.3)	19(9.4)	2.6(1.39–4.86)	**2.7(1.23–5.92)**	**0.013**
No	25(12.4)	90(44.6)	22(10.9)	1	1	1
**Prior experience in an institution where protocols/guidelines used to screen delirium**						
Yes	3(1.5)	18(8.9)	16(7.9)	3.73(1.77–7.88)	1.348(0.53–3.41)	0.528
No	25(12.4)	115(56.9)	25(12.4)	1	1	1
**Received delirium related courses educations/trainings**						
Yes	9(4.5)	54(26.7)	25(12.4)	2.15(1.19–3.9)	0.88(0.42–1.85)	0.742
No	19(9.4)	79(39.1)	16(7.9)	1	1	1
**Read guidelines/protocols related to delirium**						
Yes	6(3.0)	60(29.7)	29(14.4)	3.61(1.93–6.75)	**2.55(1.17–5.59)**	**0.019**
No	22(10.9)	73(36.1)	12(5.9)	1	1	1
**Poor collaboration in the working area**						
Yes	13(6.4)	74(36.6)	29(14.4)	1.88(1.04–3.39)	2,11(0.98–4.55)	0.057
No	15(7.4)	59(29.2)	12(5.9)	1	1	1
**Presence of work load**						
Yes	21(10.4)	110(54.5)	24(11.9)	0.47(0.24–0.94)	**0.41(0.17–0.96)**	**0.04**
No	7(3.5)	23(11.4)	17(8.4)	1	1	1
**Knowledge level**						
Poor	12(5.9)	25(12.4)	0(0.0)	0.04(0.01–0.10)	**0.04(0.01–0.13)**	**<0.001**
Moderate	15(7.4)	78(38.6)	13(6.4)	0.14(0.06–0.29)	**0.15(0.06–0.36)**	**<0.001**
Good	1(0.5)	30(14.9)	28(13.9)	1	1	1

The bold values indicates the *p*-value less than 0.05.

## 4 Discussion

Our research found that good knowledge and a positive attitude toward delirium remain lower, at 29.1 and 20.3%, respectively. Delirium is the main incidental problem in ICU settings (23–25). Physicians, nurses, and anesthetists were the direct healthcare providers for critically ill patients. Moreover, management of delirium in the ICU should be multidisciplinary approach “including physicians, nurses, and possibly other healthcare professionals (26–28). Lack of knowledge and attitude toward delirium among health professionals would affect the quality of healthcare and preventive measures (29).

This study discovered that the overall proportion of good, moderate, and poor knowledge about delirium was 29.21, 52.48, and 18.32, respectively. The proportion of good knowledge was in line with a study conducted in Iran, which found a proportion of 24.6%. However, the moderate level of knowledge was higher than in this study, with a proportion of 68.3% (29). A study done in Egypt reported a higher level of good knowledge with a proportion of 83.1% as compared to our study. However, a poor level of knowledge was comparable with this study with a proportion of 16.9% (30). A previous study that used a similar levels of knowledge category as ours found moderate and poor level of knowledge of 31 (91.2%) and 3 (8.8%), respectively, which is different from the result of this study (31). The discrepancy might be due to the small sample size they used and the fact that it was conducted only on nurse professionals.

In this study, the overall proportion of negative, neutral, and positive attitude toward delirium was 13.9 (95% CI: 9.4–19.4), 65.8 (95% CI: 58.9–72.4) and 20.3 (95% CI: 15–26.5), respectively. The proportion of positive attitudes in this study was lower as compared to reports from Egypt (66.2%) and Sri Lanka (80%) (29, 30). A study done in UK and Canada reported that most nurses had a negative attitude toward delirium in the ICU (14). However, only 13.9% of health professionals had negative attitude in our study. The possible reasons for the difference in level of attitude might be due to the categorization of the attitude section as negative, neutral, and positive in this study, while negative and positive attitudes were found in previous studies (30). The composition of study participants regarding of profession, in-service training, measurement tool, and sample size might also be possible reasons for the differences in knowledge and attitude level.

This study found that engagement in training about delirium was more likely to have an association with good knowledge, which is consistent with previous studies (30, 32, 33). A study conducted in Poland found that lack of education about delirium control had negative effect on knowledge of delirium symptoms, risk factors and complications associated with delirium in ICU patients (34). Educational interventions with inter-professional involvement increased the knowledge of clinicians about delirium in ICU (35). A focused session of delirium education for junior health professionals would increase the overall screening, investigation, and management of patients with delirium (32). Moreover, educational exposure to delirium in the form of lectures or discussions during training had a significant contribution to the improvement of the level of knowledge (36). Contrary to this fact, one study reported that the quantity of prior education about delirium did not improve the confidence, competence, and knowledge of clinicians (37).

In our study, anesthetists were more likely to have good knowledge on delirium as compared to physicians. This is supported by previous studies justifying that anesthetists spent their maximum time working in a high-level ICU (9, 26). Possibly, anesthetists might be more familiar with delirium, as it is one of the post-anesthesia complications and complications for patients with mechanical ventilators behind having good knowledge about delirium than other disciplines. Cross-sectional studies found that nurses lack the knowledge and skill to assess delirium. The difficulty of assessing delirium in intubated patients, nurses’ lack of confidence in their ability to use delirium assessment, poor organization and management, and the complexity of patients’ conditions were mentioned as potential barriers to the assessment of delirium (38).

Study participants those reporting the impossibility of changing the practice of delirium care were less likely to have good knowledge than reported the possibility to change. In agreement with this, reluctance to change delirium care practice was the possible factor that negatively affected the level of knowledge (9).

Those with a negative or neutral attitude toward delirium were less likely to have good knowledge about delirium than those with a positive attitude. These findings are supported by previous studies (29, 39). In line with this study, an earlier study reported that better knowledge was correlated with a positive attitude toward delirium (39). Health professionals who believed that the delirium screening tool would change delirium care were more likely to have a positive attitude as compared to those who did not, which is consistent with previous studies (9).

The presence of protocols or guidelines to screen for delirium in the ICU was more likely to have a positive attitude than the absence of the tools (9, 40). Similarly, a previous study identified that inadequate or non-existent use of delirium tools was one contributing factor to the low level of attitude toward delirium (41). In this study, health professionals who had read guidelines and protocols were more likely to have a positive attitude than those who had not read them. In agreement with this, earlier studies identified that reading guidelines and protocols for delirium care had a significant effect on the positive attitude of clinicians working in the ICU (32, 33, 42).

This study confirmed that health professionals those with a high work load were less likely to have a positive attitude than those with no work load. Similarly, previous studies identified that workload was the main determinant factor that negatively affected the level of attitude (9, 40).

Professionals working in the surgical ICU were less likely to have a positive attitude. Probably, the reason might be due to the lack of multidisciplinary collaboration and no proper policy on delirium care. In the surgical ICU, there should be special inter-professional collaborations with surgeons, anesthetists and nurses because multiple hands are applied on the patient’s perioperative care (43). Additionally, interrelating the manifestations of delirium with anesthesia and surgery independent of other comorbid features may lead to a negative attitude, and the patient may be left untreated (44).

Study participants with poor and moderate knowledge were less likely to have a positive attitude than those with good knowledge. This finding is in congruence with earlier study that knowledge played a significant role in improving the quality of healthcare and preventive measures, as well as the attitude toward dealing with delirious patients. Also, another study stated the positive correlation between attitude and knowledge toward delirium (29, 39).

The findings of this study implies that health professionals who have poor knowledge about delirium may have a negative attitude, and the *vice-versa*. Even though we did not accurately assess the impact on the patients’ care, it can be concluded that poor knowledge and a negative attitude possibly contribute for poor patient outcomes. Therefore, we suggest educational interventions so as to improve health professionals’ knowledge and attitude to indirectly improve the patient’s outcome. Finally, the findings of this study can form the basis for an intervention module that can address risk factors, assessment, and management of delirium in ICU.

### 4.1 The strength and limitations of the study

The possible strength of the study would be the use of primary data sources; being a multi-center study, and the research is the first of its type in Ethiopia since we did not access any published evidences, which would create motivation for researchers to do more research on similar studies. As limitations, self-administered responses may have unavoidable inaccuracies due to response bias, which could have been caused by poor recall or misunderstanding of questions and exposed to social desirability bias as respondents may over-report their attitudes.

## 5 Conclusions and recommendations

More than half of health professionals had moderate knowledge (52.48%) and a neutral attitude (65.8%) toward delirium. However, there was still some proportion of health professionals with poor knowledge (18.32%) and a negative attitude (13.9%). Only (29.21%) and (20.3%) of the study subjects had good knowledge and a positive attitude, respectively. Being anesthetist and having educations training about delirium were positively associated with good knowledge while believing delirium screening tools will change delirium care, using protocols or guidelines to screen delirium, and reading guidelines or protocols were positively associated with a positive attitude. However, it is impossible to change the practice of delirium care; negative attitudes, and neutral attitudes were delaying factors for good knowledge. Similarly, work load, poor knowledge, and moderate knowledge were hindering factors for a positive attitude. So, we recommend that concerned bodies prepare regular trainings and courses related to delirium. Also, we urge health professionals to update themselves by reading recent guidelines and protocols, as well as to prepare and use screening protocols and guidelines.

## Data availability statement

The datasets presented in this article will be made available upon request from the corresponding author.

## Ethics statement

The studies involving humans were approved by Ethical Review Committee of University of Gondar, school of medicine. The studies were conducted in accordance with the local legislation and institutional requirements. The participants provided their written informed consent to participate in this study.

## Author contributions

RA: Conceptualization, Formal Analysis, Writing – original draft. TG: Software, Writing – review & editing. WC: Writing – review & editing, Conceptualization, Formal Analysis, Visualization. DY: Writing – review & editing, Methodology, Software, Supervision, Validation. HE: Conceptualization, Writing – review & editing.
